# Detection and Analysis of Heartbeats in Seismocardiogram Signals

**DOI:** 10.3390/s20061670

**Published:** 2020-03-17

**Authors:** Niccolò Mora, Federico Cocconcelli, Guido Matrella, Paolo Ciampolini

**Affiliations:** Dip. Ingegneria e Architettura, Università di Parma, Parco Area delle Scienze 181/A, 43124 Parma (PR), Italy; niccolo.mora@unipr.it (N.M.); federico.cocconcelli@unipr.it (F.C.); guido.matrella@unipr.it (G.M.)

**Keywords:** variational autoencoder (VAE), convolutional neural network (CNN), seismocardiogram (SCG), vital sign monitoring, active assisted living (AAL)

## Abstract

This paper presents an unsupervised methodology to analyze SeismoCardioGram (SCG) signals. Starting from raw accelerometric data, heartbeat complexes are extracted and annotated, using a two-step procedure. An unsupervised calibration procedure is added to better adapt to different user patterns. Results show that the performance scores achieved by the proposed methodology improve over related literature: on average, 98.5% sensitivity and 98.6% precision are achieved in beat detection, whereas RMS (Root Mean Square) error in heartbeat interval estimation is as low as 4.6 ms. This allows SCG heartbeat complexes to be reliably extracted. Then, the morphological information of such waveforms is further processed by means of a modular Convolutional Variational AutoEncoder network, aiming at extracting compressed, meaningful representation. After unsupervised training, the VAE network is able to recognize different signal morphologies, associating each user to its specific patterns with high accuracy, as indicated by specific performance metrics (including adjusted random and mutual information score, completeness, and homogeneity). Finally, a Linear Model is used to interpret the results of clustering in the learned latent space, highlighting the impact of different VAE architectural parameters (i.e., number of stacked convolutional units and dimension of latent space).

## 1. Introduction

**Figure 1:**
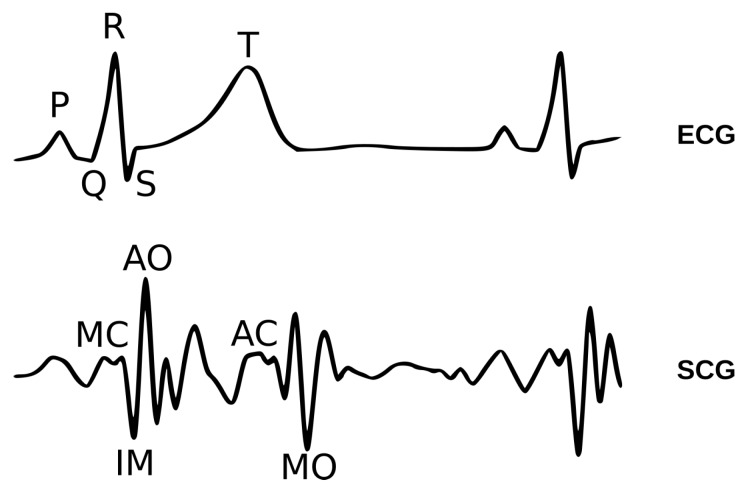
Temporal relations between ECG and SCG waveforms; fiducial points of each signal are annotated as well.

Improvements in sensing, networking, and computing technology opened up new scenarios within the Active and Healthy Ageing (AHA) realm: in particular, new technologies foster more effective monitoring, allowing for more extensive and continuous data collection and for higher dimensionality, with new kinds of data entering the monitoring scenario. For example, AAL systems [1] can now provide useful services to promote independent life of senior citizens by analyzing data collected in daily living scenarios. In some cases, an AAL system can help in making services more accessible, by compensating physical or sensory impairments with new smart devices: voice control or even Brain–Computer Interfaces have been integrated within AAL ecosystems to allow severely motor-impaired users to achieve communication and home control [2,3,4]. In addition, AAL systems can also be oriented to prevention and monitoring; in particular, the daily living patterns and activities, collected by a network of smart home sensors [5], can be analyzed by means of artificial intelligence techniques [6,7,8]: models of users’ habits can be derived, useful for detecting significant behavioral changes through time. Combining such behavioral monitoring with continuous monitoring of vital signs may provide relevant insights [9], with vital signs being contextualized and correlated to activities being actually carried out. Wrist-worn devices acquiring Heart Rate (HR) information through PhotoPlethysmoGraphy (PPG) are already a commercial reality, whereas clinical-grade heart monitoring can be achieved through standard ElectroCardioGraphy (ECG) practice. Indeed, recent improvements of embedded and low-power technologies open new possibilities to acquire heart activity information in a noninvasive and minimally obtrusive fashion. For example, by means of small MEMS (Micro Electro-Mechanical Systems) accelerometers attached to the chest wall, it is possible to record vibrations induced by cardiac activity: the study of such waveforms is the object of SCG. Indeed, analyses on SCG waveforms highlighted precise relations to specific events in the heart cycle [10]; SCG records traces of heart mechanical activity, which of course correlates to electrical activity inferred by standard ECG techniques: Figure 1 shows the temporal relation between ECG and SCG traces. SCG, in particular, allows for noticing many relevant events such as Mitral valve Opening (MO), and Closure (MC), Isovolumetric Moment (IM) contraction, Aortic valve Opening (AO) and Closure (AC). SCG thus offers great potential for heart dynamics monitoring. By precisely recognizing cardiac cycle in SCG traces, it is possible to straightforwardly evaluate, for example, HR and HR Variability (HRV) (e.g., by looking for AO/IM events). By grouping several heartbeat events into ensembles, morphological features of the waveform can be assessed. Such features are, in general, quite repetitive and consistent for a given subject, while significantly differing from one subject to others. Therefore, they can allow for biometric signature recognition or for detection of abnormal patterns, as discussed further on.

This paper presents a methodology to first detect and extract cardiac cycles directly from SCG traces. By comparison with ECG gold standard, such procedure is demonstrated to be accurate in terms of heartbeat detection and timing. This, in turn, makes it possible to precisely segment and analyze information in every single SCG heartbeat. In particular, a Variational AutoEncoder (VAE) architecture is used to extract information from the morphology of the SCG heartbeat signals. By leveraging a set of One-Dimensional Convolutional Neural Network (1D-CNN) cores, the VAE learns a complex, embedded representation of such signals. Indeed, it is shown that the proposed neural network architecture is capable of learning differences in SCG patterns between different subjects, all in an unsupervised way. In fact, good separability of the subjects is achieved by unsupervised clustering in the learned latent space. Furthermore, the performance of such clustering operation is studied, with respect to different parameters (namely, dimension of embedded space and number of cascaded convolutional units). A linear model is fitted to this purpose, quantifying the effects of such parameters.

## 2. Methods

### 2.1. Related Works

Seismocardiography is the study of precordial vibrations due to cardiac activity. Seismocardiograms are typically obtained from MEMS accelerometers, firmly attached to the subject’s chest, close to the sternum, by means of dedicated fixtures [11]. Some works explore different sensor placements over the chest wall, as reported in [12]; in [13], the impact of sensor placement on the estimation of Pre-Ejection Period (PEP, a relevant metric used in heart dynamics monitoring) is assessed. Reliable markers for cardiac activity were also found in gyroscope traces [14], a technique named GyroCardioGraphy (GCG). By combining the six degrees of freedom provided by a gyroscope and an accelerometer, the authors in [15,16] detect different systolic events for HR estimation. Both works, however, leveraged ensemble averaging techniques to improve signal-to-noise ratio. This, in turn, could reduce the timing annotation precision for single beats. In addition, many works explored the possibility of making non-contact SCG measurements. HR was estimated from clothed skin by means of an airborne pulse-Doppler ultrasound system [17]; other works use radar systems tuned at different frequency bands [18,19], microwave sensors [20], and video-based analysis [21]. However, such solutions require bulky equipment and demanding setups, not fitting the aimed AAL-oriented scenario. In the following, we therefore focus on the accelerometer approach.

Since the aimed target scenario consists of continuous monitoring of seniors users and of their daily living patterns, quasi-static, quiet conditions are likely to occur for significant time intervals during the day. During such relatively quiet intervals, accurate SCG annotation can be carried out, aiming at maximizing consistency in beat-to-beat interval measurement. This provides the AAL system with frequent and reliable assessment of heart parameters, to be integrated into behavioral patterns analysis. Monitoring heart dynamics, though, requires dedicated signal processing techniques for the detection of specific cardiac phases; reference [22] proposes an automated methodology, guided by a subject-driven calibration procedure, for extracting such events. Authors in [23] compare four different methods to compute signal envelopes for the detection of IV, AC points. The authors in [24], instead, detect AO events by first enhancing the signal with wavelet decomposition and reconstruction, followed by envelope extraction based on Shannon energy.

Besides systolic time intervals annotation, the morphology of SCG signals also carries relevant information. The authors in [25] design hand-crafted features acquired from ECG and SCG traces to detect anomalous heart activity. From a different perspective, raw and spectral features were successfully extracted from SCG traces by means of Convolutional Neural Networks (CNN), leading to personalized heart biometrics [26]. Moreover, recent work [27] extended this approach to PPG (PhotoPlethysmoGram) signals, using wearable sensors. Furthermore, authors in [28] propose a multi-resolution CNN in the wavelet domain that extracts features independent of phase shifts. Our proposed methodology, instead, leverages a 1D-CNN based Variational AutoEncoder to extract relevant information from the morphology of SCG heartbeats, previously segmented by means of an unsupervised technique; the use of VAE implies a generative model, which may prove useful, e.g., in the context of anomaly detection [29,30,31,32].

### 2.2. Datasets Description and Preprocessing

This work is based on the CEBS database [33], a public dataset (available on PhysioNet), often used for SCG benchmarking purposes. The dataset features recordings of 20 healthy subjects, resting in supine position on a comfortable, conventional single bed. The recorded signals are two standard lead I and lead II ECG channels, a respiration signal and the SCG from the subjects’ sternum. In particular, the SCG is acquired by means of a tri-axial accelerometer (SS5LB sensor by Biopac, Santa Barbara, CA, USA), with a bandwidth between 0.5 Hz and 100 Hz; only the dorso-ventral axis is available, though, since it expresses the most relevant information. The record length is approximately 50 min for each subject, totaling about 69,500 full heartbeats. Further details on the experimental setup are provided in [33].

In the proposed methodology, preprocessing steps were applied to both the ECG and SCG waveforms. First of all, the signals were downsampled from the original 5 kHz sampling rate down to 500 Hz. In fact, it is known that most of the SCG information is just contained within 1 Hz and 20 Hz [34], and that 50 Hz are adequate to capture the signal’s kinetic energy [35]. At the same time, a similar bandwidth is adequate to capture QRS peaks in the ECG signal, later used for performance assessment purposes. Limiting the bandwidth is essential in view of portable, embedded, and low-cost devices, suitable for the aimed continuous monitoring scenario. Following such considerations, both ECG and SCG signals were band-pass filtered by means of FIR (Finite Impulse Response) filters. For the ECG, passband was set between 0.5 Hz and 30 Hz, whereas SCG signals were bandpass filtered between 1 Hz and 20 Hz. All filtering operations were performed using zero-phase digital filtering techniques. This is capital for correct performance characterization, since it allows maintaining a precise phase relationship between SCG and ECG waveforms. On the other hand, conventional (non zero-phase) filtering could be applied when only the annotation phase is carried out in online, real-time way. The R peaks in the ECG, taken as ground-truth for heartbeat interval measurement, were extracted by means of the well-known Pan–Tompkins algorithm [36], and successively manually checked to ensure proper labeling.

In order to achieve scale-invariance and allow subsequent processing, individual SCG data were first normalized by means of *z-scoring*:

(1) $$x_{z}=\frac{x_{SCG}-\mu_{x}}{\sigma_{x}},$$

where $x_{SCG}$ is the bandpass-filtered SCG signal, $\mu_{x}$ its mean, and $\sigma_{x}$ its standard deviation. In addition, for the SCG morphology analysis, the isolated SCG heartbeat signals were further preprocessed by applying saturation to a ±5 range (i.e., $|x_{z}-\mu_{x}|\leq \pm 5\sigma_{x}$); an offset of 5 was added, in order to have a useful signal between 0 and 10. This is important to achieve an effective training of the convolutional VAE network, as discussed later.

### 2.3. Unsupervised SCG Segmentation

**Figure 2:**
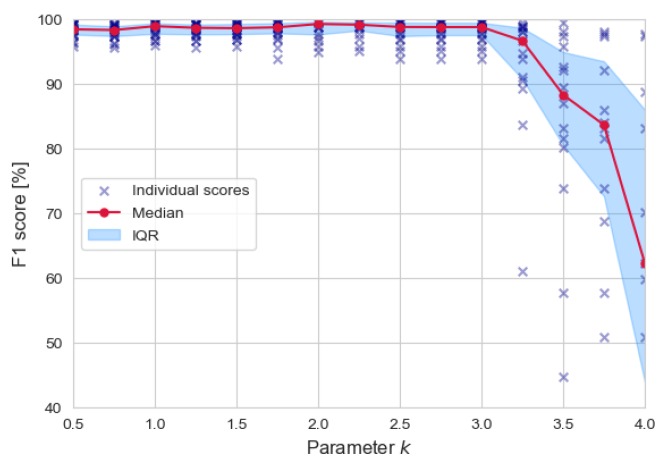
Beat detection performance (F1score=2·(Precision·Sensitivity)/(Precision+Sensitivity)) as a function of the parameter *k* in Equation (3).

An SCG heartbeat pattern consists of several peaks and valleys, which may differ in amplitude and duration, depending on the unique physiological/anatomical characteristics of each subject. To deal with such variability, a preliminary phase is introduced to ensure reliable detection and labeling of different patterns. At first, candidate heartbeats are detected by discovering local SCG energy variations, relying on the following detection signal ($x_{DET}$):

(2) $$x_{DET}\left[n\right]=\sum_{k=0}^{M-1}b\left[k\right]\cdot x_{SCG}^{2}\left[n-k\right]$$

where $x_{SCG}$ is the preprocessed SCG signal and b[k], k=0…,M−1 are the coefficients of a low-pass FIR filter; in this work, M=256 and the cutoff frequency is set to 2 Hz: this ensures proper performance on all subjects. In order to better isolate heartbeats, the $x_{DET}$ signal is further processed by means of a sliding-window filter, defined as follows:

(3) $$x_{SQR}\left(i\right)=\left\{\begin{array}{cc} 1, & \mathrm{if}\;x_{DET}\left(i\right)\geq \mu_{i:i-p}+k\cdot \sigma_{i:i-p}\\ -1, & \mathrm{otherwise} \end{array}\right.\;,$$

where $\mu_{i:i-p}$ is the average computed over the last *p* points (p=30 in this study, in order to have a sufficiently sized sample), $\sigma_{i:i-p}$ the sample standard deviation and *k* is a multiplication factor that regulates how much the sample needs to stand out against the computed window statistics. Too low values of *k* imply that more peaks are discovered, possibly including spurious ones; on the other hand, too high values of *k* might skip potential heartbeats. The optimal value of *k* was chosen by observing the beat detection performance with respect to such parameter. The F1 score was chosen as a target metric, since it fuses precision and sensitivity information into a single indicator. Figure 2 reports the output of such analysis. For lower values of *k*, the precision is slightly affected, yielding moderately lower F1 scores. On the other hand, as anticipated, high values of *k* affect the ability to detect peaks, severely impacting sensitivity and, as a result, the F1 score. Overall, an optimal value of k=2 was determined. It is worth underlining that the purpose of the $x_{SQR}$ signal is to provide a coarse indication of where heartbeats could lie (i.e., within positive periods of it). Simple, short rebounds in $x_{SQR}$ are just filtered out; on the other hand, inter-beats intervals are accepted if they fall into a pre-determined range (in this study [400 ms–1500 ms], corresponding to a heart rate within 40 and 150 beats per minute) and the relative variation is lower than 30%. In case one, the inter-beat interval results in being higher than allowed, the *k* parameter in Equation (3) is halved, and the search is performed again. A more precise localization of heartbeats (before actual annotation) is provided by looking for $x_{DET}$ maxima around positive periods in $x_{SQR}$.

Once candidate beats have been detected, an ensemble is formed from the first $n_{ENS}$ ones (this work considers $n_{ENS}$ = 20 sample traces in the ensemble), in order to form a template of each subject’s patterns. To this purpose, individual beats are incrementally aligned to each other: at first, two candidate beats are centered by aligning the extracted maxima in $x_{DET}$; then, the alignment is optimized by maximizing the cross-correlation metric on the original signal waveforms. Cross-correlation is performed over a finite set of possible delays, $\pm \tau_{S}$, with:

(4) $$\tau_{S}=s\cdot \sigma_{SQR},$$

where $\sigma_{SQR}$ represents the standard deviation of the identified positive-$x_{SQR}$ intervals length and *s* is a multiplicative coefficient: a value s=3 was determined as optimal by means of a sensitivity analysis over the full dataset. Restricting the delays to $\pm \tau_{S}$ reduces the risk of spurious misalignment and better adapts to different subjects. After alignment, a template $x_{SCG,T}$ is finally extracted by computing the median waveform of the ensemble. It is worth remarking that, by construction, such calibration procedure is performed in a completely unsupervised way, i.e., without any involvement of the ECG signal.

The SCG annotation phase follows the same steps: candidates are first identified within positive intervals in $x_{SQR}$ and successively refined according to the above criteria; then, precise time instants are marked by maximizing cross-correlation between the previously extracted template and the SCG traces in such intervals. The processing steps are summarized in Algorithm 1.

### 2.4. Convolutional Variational AutoEncoder

Variational AutoEncoders (VAE) [37] are a class of generative models that bear a resemblance to AutoEncoders (AE). Both models are structured in two components: an encoder, responsible for learning a compressed (latent) representation of input data, and a decoder, whose task is to reconstruct data from the latent space back to the original one. Both models are trained in an unsupervised way, i.e., they are fitted to compress the input and reconstruct it with the least possible error. However, unlike AE, the latent space of VAE is continuous and smooth, and this characteristic is leveraged to build the data generative model. In other words, the objective of VAE is to approximate the probability distribution of the input.
**Algorithm 1** Detection of heartbeat events.**Inputs:**$x_{SCG}$: SCG signal **Begin:**Compute $x_{DET}$ and $x_{SQR}$ according to Equations (2) and (3), respectively*Calibration*:1.Find positive $x_{SQR}$ periods such that:(a)are separated by 400 ms up to 1500 ms(b)relative variation in separation is not greater than ±30%2.Find $x_{DET}$ maxima around periods found at point 13.Compute $\tau_{S}$ from Equation (4)4.Incrementally align sample beats:(a)align waveforms around time instants found at point 2(b)refine alignment by performing cross-correlation of $x_{SCG}$ within $\pm \tau_{S}$ of the time instants found above at point 4a5.Extract SCG beat template (median)*Annotation*:6.Perform steps 1–37.Perform steps 4a–4b by aligning the template and the current $x_{SCG}$, $x_{DET}$ waveforms8.Mark the maximal alignment point as heartbeat location **Return** time instants of heartbeats


In order to make the problem treatable, VAEs use variational inference methods, i.e., they approximate the function that projects data from the original to the latent space by means of another distribution, Q(z|x), easier to sample from. Typically, such function is chosen to be a multivariate Gaussian, i.e., $Q\left(z|x\right)∼N\left(z;\mu (x),\Sigma (x)\right)$, where *z* is a variable in the latent space, and μ(x) and Σ(x) are the mean and covariance matrix of such distribution, which depend on the original input space *x*, transformed by the hidden network layers (i.e., the encoder portion). Furthermore, to impose smoothness in the latent space, a regularization term is added during learning, driving Q(z|x) towards a known prior, such as a multivariate Gaussian with null mean and unitary covariance matrix ($Q_{N}\left(z\right)∼N\left(0,I_{d}\right)$, where $I_{d}$ is *d*-dimensional unitary matrix) [37]. Such regularization term is taken as the KL divergence between Q(z|x) and $Q_{N}\left(z\right)$. Assuming a Mean Squared Error (MSE) penalty for the VAE-reconstructed input signal, the cost function $L_{VAE}$, to be optimized by gradient descent, can be written as:

(5) $$L_{VAE}=E\left\{{\left(x-\hat{x}\right)}^{2}\right\}+D_{KL}\{Q\left(z|x\right)\;||\;Q_{N}\left(z\right)\}\;$$

where *x* is the original input signal, $\hat{x}$ its reconstruction by the VAE, and the second term in the sum is the discussed regularization term.

**Figure 3:**
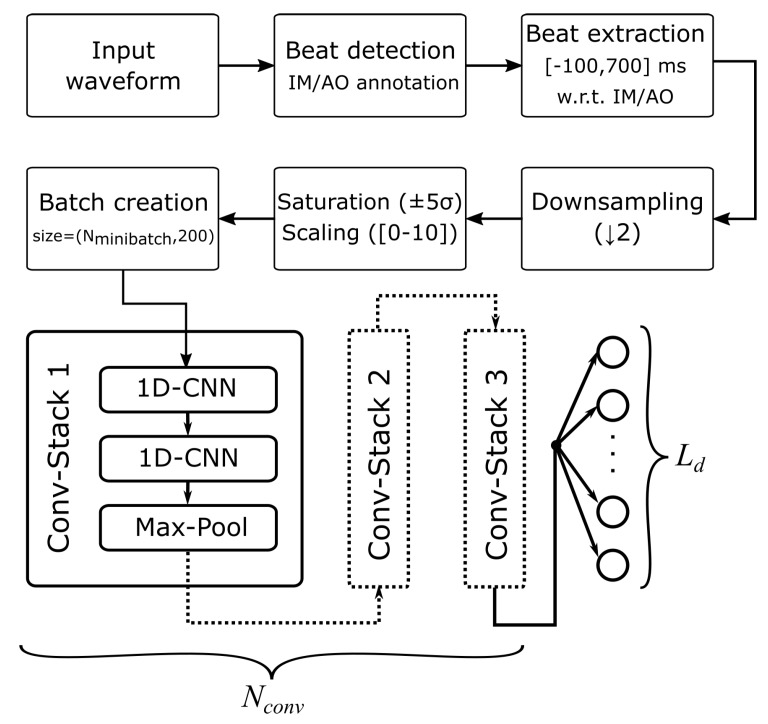
General architecture for processing the input waveforms by means of the VAE network. For clarity’s sake, only the encoder part is represented; the decoder is anti-symmetric. A convolutional stack (Conv-Stack) consists of two cascaded Conv-1D layers and a Max-Pooling one. Many Conv-Stacks can be cascaded (controlled by parameter $N_{conv}$), yielding different NN depths. The number of units in the last layer, defining the Latent Dimension $L_{d}$, is parametric as well.

In the present work, a modular Convolutional VAE (Conv-VAE) architecture is proposed for analyzing information hidden in the morphology of SCG heartbeats. In fact, the methodology presented so far allowed to detect and temporally localize single heartbeats in SCG waveforms. The results, discussed in Section 3.2.1, show that such annotation can be performed with high accuracy and temporal resolution, enabling precise SCG heartbeats’ segmentation. Such isolated beats can be studied by the proposed VAE network; however, in order to be processed by it, extracted SCG heartbeats undergo some preliminary transformation. Figure 3 shows a block-scheme of the NN architecture and the pre-processing steps applied to the extracted SCG heartbeats before being fed to the VAE. In particular, the SCG heartbeats are first cropped to a range extending from 100 ms before and 700 ms after the annotated IM-AO complex. Furthermore, in order to reduce the NN parameters, the extracted heartbeat events are downsampled by a factor of two. In this way, the 800 ms-wide SCG segments are represented by just 200 samples. As mentioned, the input waveforms are further scaled to a 0–10 range by first applying a saturation to ±5 range (i.e., $|x_{z}-\mu_{x}|\leq \pm 5\sigma_{x}$), followed by an offset of 5. In this way, inputs to the NN are homogeneous and training can be performed more effectively. Pre-processed heartbeats are then organized into mini-batches to perform training; therefore, the input size to the NN is ($N_{minibatch}$,200), where $N_{minibatch}$ is the training mini-batch size (32 in this case). The described pre-processing steps are summarized in the top part of Figure 3.

As far as the architecture of the VAE is concerned, we now introduce the encoder part; decoder description is omitted for conciseness’ sake, since the blocks are the same as the encoder, but in reverse order of application. The bottom part of Figure 3 summarizes the scheme of the encoder section: the previously extracted 200 sample-long SCG traces are fed to a modular block, from now on referred to as Conv-Stack. The block consists of the cascade of two 1D CNN layers, followed by a Max-Pooling layer that halves the equivalent signal length. Many Conv-Stacks can be cascaded, yielding a hierarchical NN structure: the number of blocks in sequence is a parameter ($N_{conv}$), whose influence on key metrics will be explored in the Results section. The output of Conv-Stacks is then fed to a dense layer, whose number of units, $L_{d}$ (i.e., the dimensionality of the latent space), is also a parameter. On the other hand, the decoder part of the VAE, which follows the encoder section, is anti-symmetric with respect to it (inverse operations), like for conventional AEs. Training is performed such that the output of the VAE (i.e., the decoder’s output) closely matches the original input, optimizing the cost function expressed by Equation (5).

## 3. Results and Discussion

### 3.1. Performance Metrics and Evaluation Procedures

In the following, we introduce several performance metrics for evaluating the two key aspects of the proposed methodology, namely:

- the ability to detect and precisely localize heartbeats, directly from SCG signals;
- the capability of learning compressed representations that reflect complex SCG morphological information.

With respect to the beat detection and annotation task, a regular heart rhythm is assumed: in this way, it is possible to compare the performance of the proposed methodology against the ECG reference (i.e., the absence of ectopic beats or rhythm disturbances in the examined records allow electrical and mechanical activity of the heart to be precisely correlated). It is worth further underlining that the mentioned calibration phase is fully unsupervised, i.e., ECG is not used in any calibration or annotation step, but it is introduced for benchmarking purposes only.

The detection of an SCG heartbeat is considered successful (True Positive, or TP) if it falls within a 100 ms-long tolerance window, around its expected location; such expected location can be estimated, for performance assessment purposes only, by collecting statistics on the delay between each individual subject’s R peaks and the IM/AO complexes in the SCG (Figure 1). On the other hand, a missed beat between two R peaks is classified as False Negative (FN); a beat outside the specified tolerance window and between two R peaks is considered as a False Positive (FP), whereas a True Negative (TN) is when no beats are found between two consecutive tolerance windows. Based on these definitions, the following metrics are introduced [38]:

- *Sensitivity*, i.e., percentage of correctly identified reference points: Sens=TP/(TP+FN);
- *Precision*, i.e., percentage of TP in all detected points: Prec=TP/(TP+FP).
- *Specificity*, i.e., percentage of TN outside the tolerance window: Spec=TN/(TN+FP).

In addition, let us define the errors between the reference ECG heartbeat intervals and the SCG ones as:

(6) $$e_{i}=t_{RR,i}-t_{CC,i},$$

where $t_{RR,i}=t_{R,i}-t_{R,i-1}$ is the *i-th* R-R interval and $t_{CC,i}=t_{SCG,i}-t_{SCG,i-1}$ is the time interval between two SCG Complexes (C–C). For convenience, let us define $t_{RR}$ and $t_{CC}$ as the time series of matched $t_{RR,i}$ and $t_{CC,i}$ intervals, whereas *e* represents the time series of errors $e_{i}$. The following performance metrics are then used [38]:

- *Error mean*$\mu_{e}=\sum_{i=1}^{N}e_{i}$, where N is the number of detected heartbeats
- *Error standard deviation*$\sigma_{e}=\sqrt{1/\left(N-1\right)\cdot \sum_{i=1}^{N}{\left(e_{i}-\mu_{e}\right)}^{2}}$;
- *Root Mean Squared Error* RMSE = $\sqrt{1/N\cdot \sum_{i=1}^{N}e_{i}^{2}}$;
- *Coefficient of Determination*$R^{2}=100\%\cdot \left(1-\sum_{i=1}^{N}e_{i}^{2}/\sum_{i=1}^{N}{\left(t_{RR,i}-\bar{t_{RR}}\right)}^{2}\right)$, where $\bar{t_{RR}}$ is the average R-R interval.

Once detected and localized, single heartbeat events in the SCG can be isolated and further processed to extract information from their morphology. In particular, this work studies the ability of different Convolutional-VAE architectures to extract meaningful compressed representation of such input data. This is, indeed, an unsupervised task: VAEs are trained to reproduce input samples, with the additional constraint of forming a smooth latent space representation. The degree of information that such NN was able to self-learn can be assessed by measuring their ability to perform unsupervised clustering in the latent space. In other words, if VAEs are properly trained, features from different subjects should be well separable in the latent space. A standard clustering algorithm like *k-means* can be used to perform unsupervised labeling. It is worth remarking again that the objective function of VAEs does not directly optimize clustering quality, but rather it minimizes the loss function of Equation (5): proper cluster separability is a secondary outcome and an indicator that relevant latent information was captured effectively. In order to evaluate the quality of cluster separation, the following main metrics were considered:

- *Adjusted Rand Score*, measuring the similarity between the unsupervised labeling and the ground truth: values close to 0 represent random labeling, whereas values close to 1 indicate good performance;
- *Adjusted Mutual Information Score*, similar to the previous one, but based on the mutual information between predicted labels and ground truth;
- *Completeness*, i.e., the fraction of members of a given class that are assigned to the same cluster. Just like the Adjusted Rand Score, a value of 1 is the theoretical maximum, achieving ideal completeness.
- *Homogeneity*, i.e., to which extent each cluster contains only members of a single class (1 indicates perfect homogeneity in cluster populations).

### 3.2. Results and Discussion

The present methodology aims at future integration of a low-cost, wearable device, suitable for multi-purpose monitoring and for adoption in practical AAL environments. In this context, the accelerometer sensor can be used to acquire multiple information, depending on actual conditions: for example, if significant motion is detected, the sensor can be used to track both quantity and quality of movement. On the other hand, during relatively quiet periods, SCG can be used to acquire information about heart activity. This continuous, multi-objective monitoring can provide useful, long term information that can enrich existing AAL services. Since heartbeat analysis is limited to quiet intervals, the CEBS database (which includes data acquired from subjects lying in bed) can be exploited to test methodologies for SCG analysis in such a quiet scenario. This condition is, indeed, representative of resting periods, especially overnight. Moreover, previous work [22] showed that similar SCG analysis methodologies can be applied to different scenarios, involving sitting users: this further extends the range of possible monitoring contexts. In this work, however, we restrict the focus on the CEBS database only, since it provides a public, trusted collection of SCG waveforms that can be used as common benchmark. Furthermore, the relatively large sample of heartbeats in the CEBS database makes it possible to build a comprehensive dataset, suitable for training deep-learning models.

In Section 3.2.1, we present and discuss the results achieved in terms of SCG heartbeat detection and annotation. High sensitivity and precision in single heartbeat detection and annotation are capital for proper posterior analysis by means of the presented convolutional VAE architecture. Indeed, Section 3.2.2 presents the results achieved by the proposed neural network in terms of complex feature learning: single heartbeats are, in fact, successfully clustered in an unsupervised way, achieving good separability of the subjects in the learned feature space.

#### 3.2.1. SCG Segmentation

**Figure 4:**
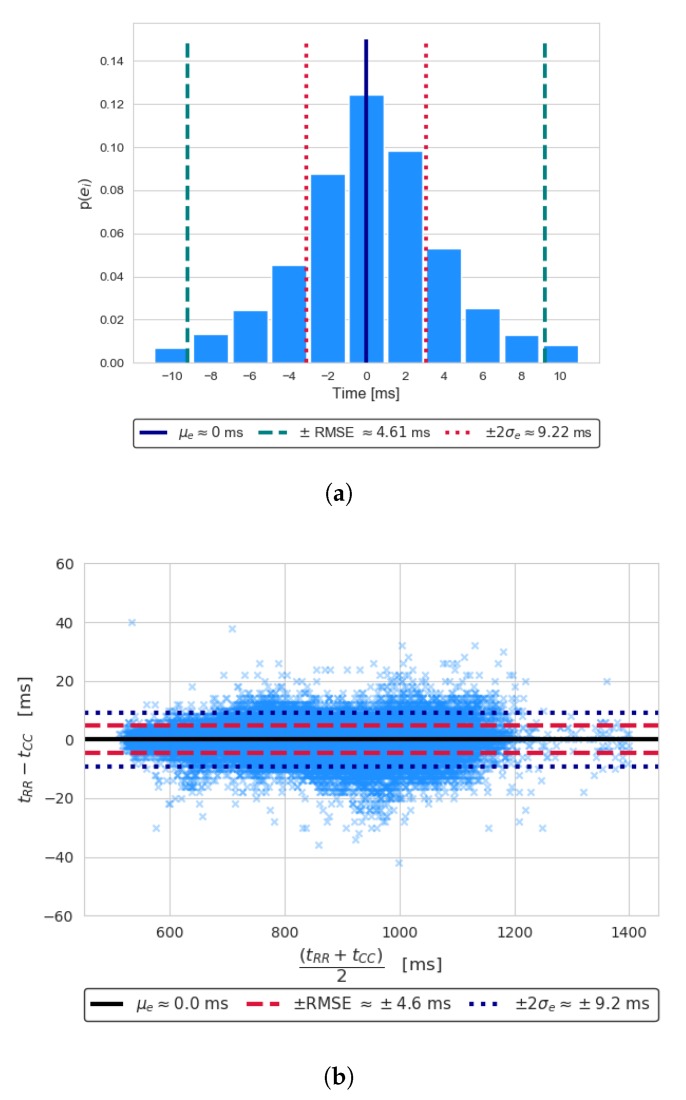
SCG events annotation performance: (**a**) error histogram, (**b**) Bland–Altman plot. Mean, RMS, and two times the standard deviation of errors are reported in the insets.

Table 1 and Table 2 show the performance scores achieved on the CEBS dataset. In particular, as reported in Table 1, good average sensitivity is achieved (98.5%) across all subjects, together with a high precision (98.6%) and specificity (98.6%): this demonstrates the ability to successfully detect the large majority of heartbeats, while preserving good immunity to false positives. In terms of consistency in time localization of such detected heartbeats, a high $R^{2}$ score was achieved (99.3%, on average): this indicates that a good agreement is obtained between ECG (i.e., reference) and SCG derived heartbeat intervals. Further insights about annotation performance are reported in Table 2, which summarizes the errors in heartbeat intervals estimation, as defined in Equation (6). More details about such scores are provided in Figure 4; in particular, Figure 4a shows an histogram plot of the errors $e_{i}$: the RMS value is reported (red dashed lines), together with the $\pm 2\sigma_{e}$ uncertainty (blue dotted line), whereas the average error $\mu_{e}$, representing systematic bias, is shown as a black solid line. It can be noticed that $\mu_{e}\approx 0$ ms, i.e., the observed bias between $t_{RR}$ and $t_{CC}$ is actually negligible; furthermore, the RMSE is contained to 4.6 ms (or $2.3\;T_{s}$, where $T_{s}$ is the sampling interval, i.e., 2 ms). Another useful visualization of $t_{RR,i}$ and $t_{CC,i}$ agreement is shown by the Bland–Altman plot of Figure 4b, which relate the measurement errors $e_{i}$ to the average of the measurements being compared, i.e., $(t_{RR,i}+t_{CC,i})/2$). This allows highlighting patterns in errors i.e., to detect dependencies with respect to the measured intervals. From inspection of such plots, errors are distributed quite evenly (as confirmed by $\mu_{e}\approx 0$): the slight apparent over-dispersion between 900 ms and 1000 ms is due to the relatively higher abundance of points in that resting HR range. Similarly to Figure 4a, the $\mu_{e}$, $\pm 2\sigma_{e}$ level and RMSE are reported: overall, good agreement can be observed.

The achieved performance metrics improve over our previous work, focused on SCG heartbeat annotation [22]. In particular, average sensitivity is greatly improved: currently, an average 98.5% score is achieved, with respect to the previous 91.4%. The standard deviation of errors, $\sigma_{e}$, is also reduced: a Mann–Whitney U-test on individual scores from the two works confirms the significance of such difference (p<0.01). Results well compare to related literature. For example, the authors in [24] achieves 94% sensitivity and 90% precision scores in detecting Aortic valve Opening (AO) events. However, such results were obtained on a subset consisting of 4585 heartbeats, which approximately represent 6.5% of the full CEBS dataset: this work, instead, does not discard any data for performance assessment. The authors in [39], instead, study the performance of SCG annotation during a lower body negative pressure test in lying position: 94% coverage is achieved over approximately 21,600 heartbeats, with an annotation time error of 9±9 ms (mean±standard deviation). In a similar setting [40], an RMSE of 40, 71, 26, 51, and 27 ms for increasing levels of lower body negative pressure; in turn, the average sensitivity in IM detection is 97.2%, 93.0%, 76.9%, 61.6%, and 65.0%, respectively. Moreover, the authors in [41] annotate heartbeats of subjects lying in bed for approximately 10 min: by fusing accelerometer and gyroscope information, they achieve high accuracy and precision scores (99.9% and 99.6%, respectively). In this case too, approximately 6.6% of data were discarded in preprocessing, to remove potential artifacts (whereas no temporal segments were discarded in our analysis). Authors also achieved a mean RMSE of 5.6 ms, with a sampling rate of 800 Hz ($\approx 4.5\;T_{s}$). The results achieved by our proposed methodology well compare to such works in literature, featuring a lower RMSE error of 4.6 ms, also considering the equivalent length in units of samples ($\approx 2.3\;T_{s}$).

#### 3.2.2. SCG Feature Learning

As previously mentioned, this work leverages the representational power of VAEs to study single SCG heartbeats. In particular, since every individual features characteristic SCG patterns, being able to recognize subjects from their waveforms imply that the information hidden in the signal morphology is properly extracted. More in detail, in order to assess whether the VAE managed to learn a compact, yet meaningful representation of the SCG heartbeats, we apply unsupervised clustering in the embedded space. Indeed, if the VAE is successfully trained, it should recognize different patterns in the waveforms. This can be tested by monitoring the clustering quality; in other words, if the clustering operation yields classes that are composed of just a single individual’s waveforms, the learned representation is successful in compressing the SCG morphological information. It is worth mentioning that, in order to perform rigorous measurement of the performance, the dataset was split into separate training, validation, and test set (with a 70%, 15% and 15% ratio, respectively). In the following, only the test set performance is reported, so that unbiased estimates are presented.

**Figure 5:**
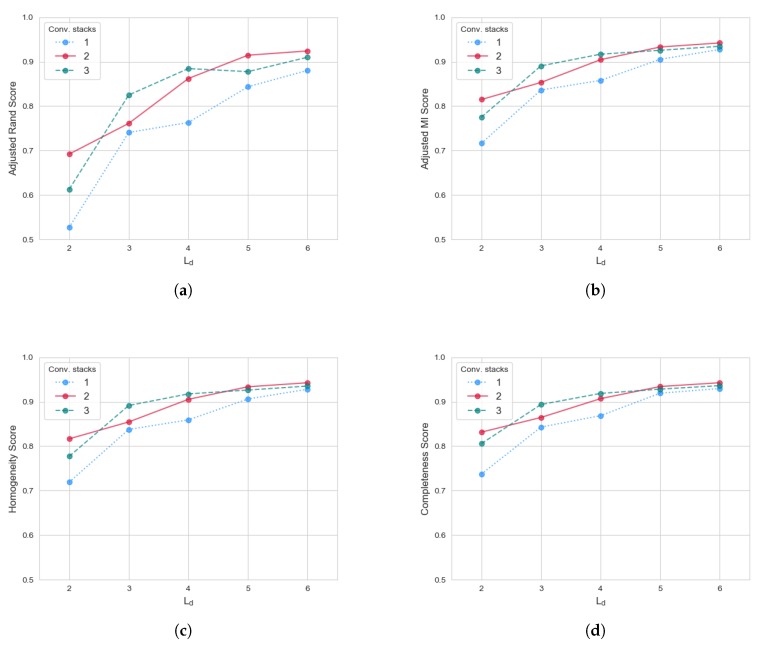
SCG deep clustering performance with respect to latent dimension $L_{d}$: (**a**) Adjusted Rand Score, (**b**) Adjusted Mutual Information Score, (**c**) Homogeneity, and (**d**) Completeness. Performances for a different number of conv-stacks are superimposed, using different colors and line styles.

Figure 5 shows the performance achieved by different VAE architectures in terms of Adjusted Random Score (Figure 5a) Adjusted Mutual Information Score (Figure 5b), Completeness (Figure 5d) and Homogeneity (Figure 5c). In particular, each score is plotted against the VAE latent dimension ($L_{d}$), whereas performance traces of differently deep VAEs (i.e., number of cascaded Conv-Stacks) are superimposed on the same plots.

Results show that scores well above 90% can be achieved in all metrics in the best parameter settings. This suggests that clusters are very well represented and populated. In particular, it can be observed that VAEs with just one convolutional stack exhibit the lowest overall performance: such relatively shallow network are not well suited at extracting complex and meaningful patterns from the input data. On the other hand, VAEs with two convolutional stacks tend to perform better at the extremes of the explored $L_{d}$ range, whereas VAEs with three convolutional stacks perform better in mid-sized $L_{d}$ range. A possible explanation is that, with moderate dimensional latent space, the triple stacked convolutional units better summarize the data in lower dimension (they perform an extra max-pool operation, with respect to their double stack counterparts). On the other hand, when the latent space is more expressive (i.e., more dimensions are added), the data are better mapped when no third max pooling operation is performed, preserving that extra information; the same could be true for lower-dimension latent space ($L_{d}=2$), with double stacked VAEs being the right compromise between performing meaningful convolutional transformation while compressing information. Nonetheless, performance difference between differently deep VAEs tend to get lower as $L_{d}$ increases.

In order to quantitatively interpret the results of Figure 5, the following Linear Model is introduced:

(7) $$y_{perf}=\beta_{0}+\beta_{1}\cdot \sqrt{\Delta L_{d}}+\beta_{2}\cdot \Delta N_{conv}\;$$

where $y_{perf}$ is the target performance metric (Adjusted Rand, Mutual Information, Completeness, and Homogeneity scores), $\beta_{0}$ is an intercept term, $\beta_{1}$ is the coefficient modeling the effect of an increase in the latent space size ($L_{d}$), and $\beta_{2}$ quantifies the effect of stacking more convolutional layers. In particular, such covariates represent the effect of an increase with respect to the baseline case, represented by the intercept term (i.e., $L_{d}=2$ and $N_{conv}=1$). In other terms, the model tries to quantify the performance improvements achieved by unit increase of the square root of VAE latent dimension and unit increase of the VAE depth, with respect to the baseline case.

Table 3 details the results of model fitting, for each performance metric. In addition, the model $R^{2}$ score is reported: the high values of such metric indicate that most of the performance data variance has been captured properly, thus allowing to interpret each factor effect more rigorously. In particular, it can be noticed that the $\sqrt{\Delta L_{d}}$ factor contributes the most in improving all performance scores, with respect to the baseline (i.e., $N_{conv}=1$, $L_{d}=2$). This effect is more important at lower latent space dimension, as confirmed by the proportionality to the square root of $\Delta L_{d}$: further improvements in scores proceed with sub-linear behavior, with respect to increasing latent space dimension. On the other hand, increasing the number of convolutional stacks ($\Delta N_{conv}$) also has a positive impact. However, the relative effect is lower: approximately, the $\sqrt{\Delta L_{d}}$ factor is four times stronger.

Overall, the presented results indicate that the VAE network is able to learn meaningful compressed representations of the input SCG heartbeat complexes. Table 4 reports the best scores achieved by the VAE network, with respect to the number of convolutional stacks. In particular, the VAE with $N_{conv}=2$ is the best performing one, with scores well above 0.94 (with 1.0 being the maximum value); such scores are remarkable, considering that the dataset features 20 different subjects (i.e., labels), significantly challenging unsupervised clustering. The high clustering quality demonstrates that subject-specific features are well extracted in such latent space, thus enabling to implement higher-level services such as biometric recognition or anomaly detection, based on the information encoded by the VAEs.

## 4. Conclusions

This paper presented an unsupervised methodology to analyze SeismoCardioGram signals. Starting from raw accelerometer data, SCG heartbeat complexes have been extracted and annotated, using a two-step procedure. An unsupervised calibration procedure was added, to better adapt to different user patterns. Results show that the performance scores achieved by the proposed methodology improve over related literature, both in terms of beat detection accuracy, as well as lower RMS error in heartbeat intervals estimation (with ECG taken as gold standard).

Such annotation performance makes it possible to reliably extract SCG heartbeat complexes, whose morphological information is further processed by means of Convolutional Variational AutoEncoder networks, aiming at extracting compressed, meaningful representation. In particular, this work presented a modular Convolutional VAE architecture that can be shaped and customized based on different parameters, including depth (number of stacked convolutional units) and latent space dimension. After unsupervised training, the VAE network was able to recognize different signal morphologies, associating each user to its specific patterns with high accuracy, as indicated by specific performance metrics (including adjusted random and mutual information score, completeness, and homogeneity). Finally, a Linear Model was used to interpret the results of clustering in the learned latent space, highlighting the impact of different VAE architectural parameters.

The proposed methodology can be useful in continuous monitoring scenarios, including frequent, non-intrusive acquisition of vital signs. For example, using the presented SCG segmentation methodology, individual beats can be isolated (without concurrent ECG) and analyzed with the present VAE architecture, making it possible to implement anomaly detection techniques in the latent space: this effectively enhances information that smart environments can gather on users’ wellbeing [5].

## Figures and Tables

**Table 1:** Main SCG annotation performance: individual scores.

Metric	Score
Sensitivity	98.5% (±1.2% std. dev.)
Precision	98.6% (±1.2% std. dev.)
Specificity	98.6% (±1.2% std. dev.)
R^2^	99.3% (±0.6% std. dev.)

**Table 2:** Main SCG annotation performance: population-wide scores.

Metric	Score
$\mu_{e}$	≈0 ms
RMSE	4.61 ms
$\pm 2\sigma_{e}$	9.22 ms

**Table 3:** Results of Linear model fitting of Equation (7).

Metric	Intercept	$\sqrt{\Delta L_{d}}$ Factor	$N_{\mathrm{conv}}$ Factor	$R^{2}$ [%]
Adjusted Rand	0.582	0.15	0.036	90.9
Adjusted Mutual Information	0.752	0.085	0.020	91.6
Completeness	0.774	0.075	0.019	90.9
Homogeneity	0.754	0.084	0.020	91.6

**Table 4:** Best metric scores for different convolutional stacks.

Metric	$N_{\mathrm{conv}}=1$	$N_{\mathrm{conv}}=2$	$N_{\mathrm{conv}}=3$
Adjusted Rand	0.908	0.955	0.930
Adjusted Mutual Information	0.915	0.944	0.922
Completeness	0.917	0.945	0.924
Homogeneity	0.917	0.945	0.924
